# A Cross-Sectional Serological Study to Assess the Prevalence and Risk Factors of Anaplasmosis in Dromedary Camels in Punjab, Pakistan

**DOI:** 10.3390/vetsci11120657

**Published:** 2024-12-16

**Authors:** Muhammad Zaeem Abbas, Muzafar Ghafoor, Muhammad Hammad Hussain, Mughees Aizaz Alvi, Tariq Jamil, Muhammad Sohail Sajid, Munazza Aslam, Ali Hassan, Shujaat Hussain, Mian Abdul Hafeez, Muhammad Irfan Ullah, Iahtasham Khan, Khurram Ashfaq, Ghulam Muhammad, Katja Mertens-Scholz, Heinrich Neubauer, Hosny El-Adawy, Muhammad Saqib

**Affiliations:** 1 Department of Clinical Medicine and Surgery, Faculty of Veterinary Science, University of Agriculture, Faisalabad, Pakistan; zaeemabbas45@gmail.com (M.Z.A.); muzafar1512@gmail.com (M.G.); mughees.alvi@uaf.edu.pk (M.A.A.); aslammunazza3@gmail.com (M.A.); dralihassan635@gmail.com (A.H.); drkhurramashfaq33@gmail.com (K.A.); profdrgm54@gmail.com (G.M.); 2 Department of Animal and Veterinary Sciences, College of Agricultural and Marine Sciences, Sultan Qaboos University, Muscat, Oman; m.hussain1@squ.edu.com; 3 Institute of Bacterial Infections and Zoonoses, Friedrich-Loeffler-Institut, 07743 Jena, Germanykatja.mertens-scholz@fli.de (K.M.-S.); heinrich.neubauer@fli.de (H.N.); 4 Department of Parasitology, Faculty of Veterinary Science, University of Agriculture, Faisalabad, Pakistan; drsohailuaf@hotmail.com; 5 Faculty of Veterinary and Animal Sciences, PMAS Arid Agriculture University, Rawalpindi, Pakistan; drshujaat@uaar.edu.pk; 6 Department of Parasitology, University of Veterinary and Animal Sciences, Lahore, Pakistan; abdul.hafeez@uvas.edu.pk; 7 Department of Pathobiology, Faculty of Veterinary Sciences, Bahauddin Zakariya University, Multan, Pakistan; irfanullahvet@gmail.com; 8 Department of Clinical Sciences, College of Veterinary and Animal Sciences, Jhang, University of Veterinary and Animal Sciences, Lahore, Jhang 35200, Pakistan; iahtasham.khan@uvas.edu.pk; 9 Institute for Infectious Diseases and Infection Control and Center for Sepsis Care and Control (CSCC), Jena University Hospital, Am Klinikum 1, D-07745 Jena, Germany; 10 Faculty of Veterinary Medicine, Kafrelsheikh University, Kafr El-Sheikh 33516, Egypt

**Keywords:** anaplasmosis, cELISA, risk factors, dromedary camels, Pakistan

## Abstract

Anaplasmosis is an infectious disease transmitted by ticks and caused by obligate intracellular pathogen of belonging to genus *Anaplasma* Infections of one-humped camels (*Camelus dromedarius*) and llamas (*Lama glama*) have been reported previously. The aim of this study was to investigate the seroprevalence and risk factors of anti-*Anaplasma* spp. antibodies in *Camelus dromedarius* of the Punjab, Pakistan. A cross-sectional study was conducted during 2017–2018 to study the seroprevalence of anaplasmosis in *Camelus dromedarius* of 13 districts in Punjab province of Pakistan and to assess the associated risk factors including age, breed, gender, body condition score, tick infestation, location, season and management type. Serum samples from 728 camels (433 females and 295 males) were examined for anti-*Anaplasma* antibodies using a commercially available competitive enzyme-linked immunosorbent assay (cELISA) test kit. A univariable analysis was conducted and extended to multivariate logistic regression to find potential risk factors associated with the disease. Overall, the seroprevalence of anti-*Anaplasma* antibodies was 8.5% (8.5%, CI 6.6–10.8) with 62 positives in 728 camels. The highest seroprevalence was recorded for camels of the Central Punjab districts (16.1%, CI 11.5–21.7) followed by those of the Northwestern (5.4%, 2.8–9.3) and Southern Punjab (5.2%, 2.9–8.4) districts (*p* < 0.001). Multivariable analysis showed that location (Central Punjab: OR 2.78, *p* = 0.004), season (summer: OR 7.94, *p* = 0.009), body condition score (BCS 2: OR 14.81, *p* = 0.029) and tick infestation (OR 38.59, *p* < 0.001) are potential risk factors in the corresponding camel populations. The results showed that the camel population in Pakistan is seropositive for *Anaplasma* spp. The geographical zone, season, body condition and tick infestation were identified as significantly associated risk factors for seroprevalence of anaplasmosis in dromedary camels. To the best of our knowledge, the results of this current study provide the first evidence of exposure of camels to anaplasmosis in Pakistan. Molecular investigations in the future are highly recommended to determine the dynamics of the disease in camels.

## Simple Summary

Anaplasmosis is a disease affecting animals like camels. This study aimed to understand how common this disease is in one-humped camels in Punjab, Pakistan, and to identify factors that increase the risk of infection. Researchers tested blood samples from 728 camels across 13 districts between 2017 and 2018, finding that 8.5% had been exposed to *Anaplasma*. The highest rates of infection were found in Central Punjab, particularly among camels with a poor body condition and those infested with ticks. This study concludes that the location, season, body condition and tick infestation significantly influence the risk of anaplasmosis in camels. This research is valuable to society as it provides the first evidence of the disease in camels in Pakistan, highlighting the need for better management and control of tick populations to protect these animals and improve livestock health. Future studies are encouraged to further explore the disease’s dynamics in camels.

## 1. Introduction

Anaplasmosis, a tick-borne disease of animals, is caused by the obligate intracellular bacteria of the genus *Anaplasma* (*Rickettsiales*: *Anaplasmataceae*). All species replicate within cells of the hematopoietic linage [1]. The genus *Anaplasma* includes, but is not limited to, the following species: *A. marginale*, *A. centrale*, *A. ovis*, *A. platys* and *A. phagocytophilum*. Recent research detected *A*. *marginale*, *A*. *platys*, *A*. *phagocytophilum* and *Candidatus Anaplasma camelii* as responsible for anaplasmosis in dromedaries [2,3].

A variety of hard-tick species, i.e., *Ixodes*, *Dermacentor*, *Rhipicephalus* and *Hyalomma,* are known to transmit the pathogen to susceptible hosts [4]. *Anaplasma* species are mechanically transmitted by different species of tabanids and midges [5]. The use of contaminated needles and surgical instruments could be responsible for transmission [6]. Clinically recovered domestic ruminants (cattle, sheep and goats) and wild mammals may also act as reservoirs for anaplasmosis [7]. *Anaplasma* species enter the bloodstream after the tick’s bite, replicate in erythrocytes (RBCs) and, finally, cause bursting of infected cells [8]. After infection, 15% of RBCs may be infected, but this depends on the strain type, the host’s immune response and the dose of the infection. Clinical disease may become obvious when more than 15% of RBCs are affected [9]. The clinical course of anaplasmosis can be acute, subacute or chronic. Acute anaplasmosis manifests as fever, anemia, jaundice, decreased production, i.e., a rapid decline in milk production and reduction in weight, abortion, occasionally diarrhea, and excitability [10]. Once infected, cattle may become lifelong carrier hosts, which maintain the infection in endemic regions [11].

Anaplasmosis in camels is usually associated with *Anaplasma* strains related to *A. platys*, but there are reports of camels positive for *A. phagocytophilum* or *A. ovis*. An infection in camels is generally subclinical or detected as co-infection [12,13]; in contrast, anaplasmosis may exacerbate at any time and clinical disease is favored by drought/weather stress and poor nutrition [14].

Anaplasmosis is considered endemic in the tropics and subtropics and has been reported in temperate regions of the world [15]. Several studies have reported the prevalence, distribution, and characterization of *Anaplasma* species among livestock (cattle, buffalo, sheep and goats) in Pakistan [16,17]. The disease has been linked to vectors, abundance of bacterial population, management system (grazing) and bad hygiene [18].

Stained blood smear microscopy is a conventional laboratory and field diagnostic method for the detection of animals suspected of having anaplasmosis but needing confirmation by serological testing using a competitive ELISA or PCR as recommended by the World Organization of Animal Health (WOAH) [10]. Several PCR-based tests are available as reliable tools for the detection and differentiation of *Anaplasma* species. However, the competitive ELISA (cELISA) with a sensitivity of 96% sensitivity and specificity of 95% has been widely used for serological diagnostic purposes of anaplasmosis. It is easy to perform and has low costs, but it cannot differentiate between causative *Anaplasma* species [19,20].

Pakistan is an agricultural-economy-based country where livestock play an integral role in the agriculture sector [21]. Camels are important farm animals in arid ecosystems as a source of draft power, food (milk and meat), capital, hides and hair. Additionally, camels act as show animals and status symbols for institutions and families. Pakistan is home to 1.1 million dromedary camels (*Camelus dromedarius*) [22]. Like other domestic animals, camels are susceptible to various infections. Ticks infestation and tick-borne diseases (TBDs) pose a dangerous threat to the health, production, reproduction and working efficiency of camels [23]. Although there have been few reports on the occurrence of anaplasmosis in camels [24,25], epidemiological studies have not been well-reported and disease may likely be neglected due to aysmptomatic infection and unknown economic losses. There is a dire need to carry out a systematic epidemiological survey to plan disease prevention and control strategies. Therefore, the present study was conducted to investigate the seroprevalence of anaplasmosis and associated risk factors in the camel population of the province of Punjab, Pakistan.

## 2. Materials and Methods

### 2.1. Sampling Areas, Meteorological Characteristics and Ethical Consideration

This cross-sectional serologic study was conducted on camel populations from August 2017 to December 2018 in 13 districts from Central, Southern and Northwestern Punjab province of Pakistan.

The geographic coordinates, climatic conditions and camel population of the selected districts are shown in Table 1.

Key ethical considerations were addressed, including ensuring humane handling of the animals to minimize stress and discomfort during sample collection. Informed consent was obtained from camel owners, with us clearly explaining this study’s purpose, procedures and potential risks, along with protection of the confidentiality of participants’ identities and data. The research had scientific integrity, with accurate data collection and reporting of results. This study complied with local ethical guidelines regarding animal research, and it was approved by the Directorate of Graduate Studies, University of Agriculture, Faisalabad, Pakistan.

### 2.2. Sampling Frame and Sample Collection

The population of camels in each sampled district is presented in Table 1. The total of the target population was 22,943 heads. The required sample size of 717 was calculated according to the available population estimates of camels in the sampled districts. However, it was not possible to strictly adhere with the sampling plan because of the nomadic nature of camel herds—they keep on moving in search of available pastures. There are only a few permanent camel farms/holdings available in arid districts like Bhakkar, Khushab and Layyah. Therefore, it was not possible to adhere to the minimum sample size required in these districts. The camel owners and communities were contacted in each district and were requested to participate in this study. All the available camels at a sampling location were assigned a number and marked with a temporary marker. Random selection was performed using Epitools (https://epitools.ausvet.com.au/randomnumbers (accessed on 12 January 2019)).

The sample size was calculated for simple random sampling with an expected prevalence (P_exp_) of 50% (unknown status), with confidence limits of 95% and a desired absolute precision of 5% [26]. The sample size was increased by 10% to cover expected losses during transport and handling. A minimum number of 717 camels to be sampled in the selected districts was calculated. Finally, a total of 728 (433 females and 295 male) camels were randomly sampled from 13 districts in the province of Punjab, Pakistan.

Blood was collected in 4 mL, gel-clot and EDTA-coated vacuum vials. Data on animals (age, breed, gender, body condition score, tick infestation and location) along with season and management type were recorded using a questionnaire at the time of sampling. Animals were categorized into four age groups: ≤3 years (n = 202), 3.1 to 7.0 years (n = 330), 7.1 to 10 years (n = 100) and >10 years (n = 96) of age. Sera were harvested by centrifugation and stored at −40 °C until testing.

### 2.3. Serological Screening

Antibodies against *Anaplasma* spp. in sera were detected by using the commercially available *Anaplasma* Antibody Test Kit, cELISA (Veterinary Medical Research and Development Inc., Pullman, WA, USA), according to the manufacturer’s instructions [27,28,29]. An ELISA reader (Bio-Rad Laboratories, Inc., Hercules, CA, USA) was used to measure the optical density (OD) at a 650 nm wavelength. Samples with ≥30% inhibition were considered positive and samples with <30% inhibition were considered negative. The percent inhibition was calculated using the formula: % I = 100 [1 − (Sample OD/Negative control OD)].

### 2.4. Statistical Analysis

The data were analyzed by using Statistical Package for Social Services (IBM SPSS version 20.0, IBM, New York, NY, USA). Seroprevalence (%) and 95% exact binomial confidence intervals (CI) were calculated, A univariable analysis was conducted using logistic regression (LR), and the odds ratio (OR) was calculated. The analysis was extended to multivariate LR to find potential risk factors associated with the disease. The goodness of fit for the LR model was assessed by Hosmer–Lemeshow (HL) statistics, which observed whether or not the observed event rates matched expected event rates [30]. All variables with a *p*-value < 0.20 were included in the initial model, and confounders were removed at each step until all significantly associated (*p* < 0.05) variables remained in the final model [31]. Pairwise correlation tests to the response variables addressed multicollinearity and model overfitting. All variables with *p* < 0.2 were added to the initial multivariable model. Variables with *p* > 0.05 were removed at subsequent steps (backward elimination) until a most parsimonious model was achieved. The values of the Hosmer–Lemeshow test and Nagelkerke’s R square were used to assess the model fit.

## 3. Results

**Figure 1:**
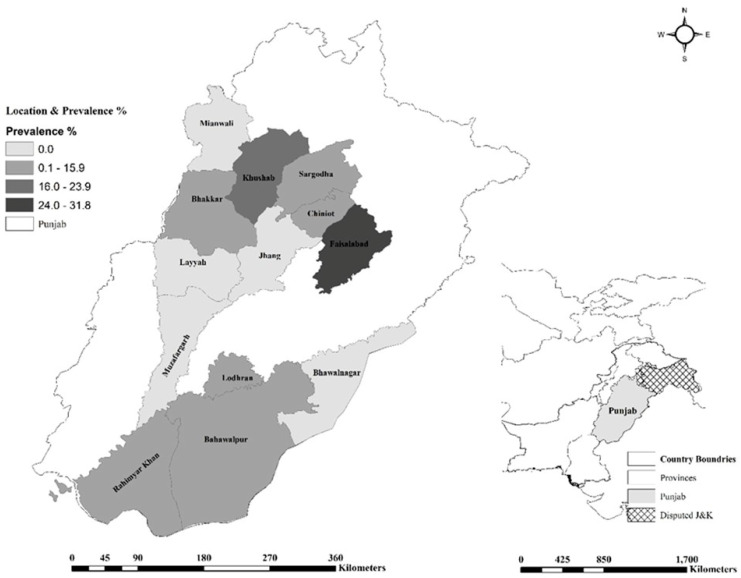
Distribution of anaplasmosis in dromedary camels of different districts of the province Punjab, Pakistan; J&K: Jammu and Kashmir.

Out of 728 collected serum samples, 62 (8.5%, CI 6.6–10.8) were found positive for anti-*Anaplasma* antibodies. Seropositive camels belonged to eight different districts and ranged from 31.8% in Faisalabad to 1.8% in Chiniot, Sargodha, and Bahawalpur districts, *p* < 0.001 (Figure 1; Table 2 and Table 3).

Higher seroprevalence was recorded in camels of the Central Punjab districts, Pakistan (16.1%, CI 11.5–21.7), followed by those of Northwestern (5.4%, 2.8–9.3) and Southern districts (5.2%, 2.9–8.4), *p* < 0.001 (Table 4). Tick infestation was recorded for 109 (14.9%) of the sampled camels. Most sampled camels belonged to the Berella breed (n = 341, 46.8%) followed by non-described breeds (n = 201, 27.6%) and the Marecha breed (n = 186, 25.5%). All animals were apparently healthy and further categorized into three different body condition score (BCS) groups: BCS ≤ 2 (n = 12, 1.6%), BCS 3 (n = 393, 53.9%) and BCS ≥ 4 (323, 44.4%) (Table 3).

Analysis indicated that camels of Central Punjab districts (OR 3.5, CI 11.5–21.7) were significantly (*p* < 0.001) more likely to test positive as compared to those in the Southern Punjab districts (Table 2). The seroprevalence (9.7%, CI 7.5–12.3) and odds (OR 5.8, CI 1.40–24.11) for testing positive were significantly (*p* = 0.015) higher in the camels sampled during summer. Higher values were detected in the non-descript camel breeds (8.9%, CI 5.4–13.8) as compared to the Berella and Marecha breeds, *p* = 0.952. Seroprevalence and odds values for testing positive were highest in camels of 3.1–7.0 years of age (11.2%, OR 2.7 CI 1.28–5.74) followed by those >10 years (10.4%, OR 2.5 CI 0.98–6.36) and 7.1 to 10 years (6.0%, OR 1.4 CI 0.47–3.96) of age, *p* = 0.403. There was no statistically significant difference in seroprevalence (8.5%) between male and female animals, *p* = 0.973. The group of camels with BCS ≤ 2 had the highest seroprevalence (66.7%, CI 34.9–90.1) and those animals were significantly (*p* < 0.001) more likely (OR 32.0, CI 8.84–115.86) to test positive for anaplasmosis. Significantly (*p* < 0.001) higher seropositivity (46.8%, CI 37.2–56.6) and odds (OR 48.6, CI 24.01–98.37) for testing positive were detected in tick-infested camels (Table 3).

Multivariable logistic regression model was applied to all significant variables (*p* < 0.05) to predict the association between prevalence of anaplasmosis and different risk factors (Table 4).

Breed, sex and age were dropped in the subsequent steps. The final multivariable analysis showed that location (central Punjab: OR 2.78, *p* = 0.004), season (summer: OR 7.94, *p* = 0.009), BCS (BCS 2: OR 14.81, *p* = 0.029) and tick infestation (OR 38.59, *p* < 0.001) were the variables associated with the higher prevalence of anaplasmosis in the sampled camel population. The values of the HL test (χ^2^ = 4.383, df = 5, *p* = 0.496) and Nagelkerke R Square (0.516) indicated that this was a good model to predict *Anaplasma* sp. seropositivity in the sampled camel population.

## 4. Discussion

Anaplasmosis is one of the important rickettsial livestock diseases in Pakistan [13,15,32,33,34,35,36]. The mainstay in the diagnosis of anaplasmosis in animals remains the microscopic identification of Giemsa-stained jugular/peripheral blood smears coupled with molecular detection techniques, e.g., conventional and/or real-time PCRs [10]. Moreover, asymptomatic carriers are difficult to diagnose by microscopy and PCRs as the number of *Anaplasma* circulating in the blood can be very low. Hence, serological techniques are an appropriate technique to determine exposure to *Anaplasma* sp. by detecting specific antibodies. Nevertheless, the seroconversion time (window period) has to be considered when declaring animals to be disease-free or acute anaplasmosis has to be considered [37]. Major surface protein 5 (*MSP5*) is a highly conserved marker in all *Anaplasmae*, and hence a good diagnostic antigen [38]. The cELISA using recombinant *MSP5* thus provides a highly sensitive and specific result but may cross-react with anti-*Ehrlichia* antibodies [39]. Keeping in view this scenario and previous validation studies, a commercial cELISA kit was used to determine the seroprevalence of anaplasmosis in camel sera collected from Punjab, Pakistan [10,20].

In the present study, the overall prevalence was 8.50%, which was a finding similar to previous studies of *Anaplasma* infections in camels from Egypt and Iran with seroprevalences of 10.7% and 6%, respectively [40,41]. Other studies conducted in Nigeria, Egypt and Iran reported higher seroprevalences of 20.3%, 18.6% and 17.14%, respectively [12,42,43]. Owing to the variability of serological tests used for anaplasmosis in camels, an objective comparison between this and previously published studies is difficult.

Prevalence was found to be higher in those areas where tick infestation was also high. The variation in seroprevalence might be due to different physical (topographical conditions), temperature, moisture, biological (cattle distribution and vector infestation) and socioeconomic factors (pasture disruption) [44]. Owing to the lack of comprehensive systemic seasonal sampling, the high incidence of anti-*Anaplasma* antibodies during summer may not represent true seasonal prevalence estimates. This limitation in sampling relates to the nomadic nature of camel populations in Pakistan.

Camels of 3.1–7 years of age and >10 years of age were found to be at higher risks as compared to other age groups in this study. These findings are in accordance with previous studies that reported a direct relation of infection with the age of the animals [45,46]. Younger animals may be protected because of colostrum antibodies from an immune dam [47]. The higher seroprevalence in older animals may be also attributed to poor management, movement from one place to another and heavy stress during transport [34].

The sex of the animals was found to be statistically non-significantly (*p* > 0.05) associated with a positive serological test, with a significantly higher prevalence in female camels reported [12,48]. A higher prevalence of anaplasmosis in camels during the summer season (9.7%) agrees with the findings of a study conducted to determine the risk factors associated with seroprevalence of *Anaplasma marginale* among cattle in Sargodha, Khushab and Rawalpindi districts of the Punjab, Pakistan during September 2009 to August 2010 [32]. Although the reference study is about *A. marginale,* and camel and cattle are the different species, the result correlation corroborates the high activity of the ticks during the summer season. Endemicity of the disease has been found throughout the year; however, infection becomes epidemic during the hot and humid months of summer because of the higher prevalence of tick vectors in this season [49,50]. Iranian investigators reported that increased contact among infested camels and other farm animals and the presence of ticks increase the risk of such infections [51]. Moreover, female animals have been found to be preferred hosts compared to males in tick-borne diseases [52,53,54].

The disease was significantly (*p* < 0.05) associated with camels having low BCS. An Ethiopian study documented that anaplasmosis is a major health and management problem in livestock, where a high tick infestation with poor body conditions was registered [52].

## 5. Conclusions

The seroprevalence of anaplasmosis in the camel population in Pakistan is significantly associated with four risk factors: geographical zone, season, poor body condition and tick infestation. Future studies must encompass the uninvestigated provinces of Pakistan to ascertain the prevalence of the disease in those areas. Furthermore, in the future, molecular detection and genotyping of *Anaplasma* species are highly recommended to better comprehend the phylogeny.

## Figures and Tables

**Table 1:** Coordinates and climatic characteristics of the study districts of Punjab provinces.

Punjab Province	Districts	Coordinates		Elevation from Sea Level (ft)	Temperature (°C)				Camel Population (Head)
		Lat. (°N)	Long. (°E)		Summer		Winter		
					Max.	Min.	Max.	Min.	
Central	Faisalabad	31°27′	73°8′	607	39	27	21	6	687
	Chiniot	31°43′	72°58′	597	40	27	19	6	663
	Jhang	31°16′	72°19′	515	41	28	19	6	1265
Northwestern	Mianwali	32°35′	71°32′	705	40	26	19	5	1886
	Bhakkar	31°37′	71°3′	561	41	28	19	6	5310
	Khushab	32°18′	72°17′	600	40	27	19	5	3712
	Sargodha	32°5′	72°40′	620	40	27	19	5	774
Southern	Bahawalnagar	30°0′	73°15′	511	41	28	20	4	681
	Layyah	25°20′	55°22′	479	41	29	19	7	3155
	Muzaffar Garh	32°7′	80°3′	390	42	28	20	7	1687
	Bahawalpur	29°21′	71°41′	370	41	29	19	6	1078
	Rahim Yar Khan	28°35′	77°14′	272	41	28	23	5	1921
	Lodhran	29°32′	71°37′	377	41	28	20	7	115

Lat. = Latitude; Long. = Longitude; Max. = Maximum; Min. = Minimum.

**Table 2:** Univariable analysis for *Anaplasma* sp. seropositive camels and the ecological zones of Punjab, Pakistan.

Zone	District	Camel Population	Positive/Tested	Prevalence % (95% CI)
Central Punjab	Chiniot	663	1/55	1.8 (0–9.7)
	Faisalabad	687	34/107	31.8 (23.1–41.5)
	Jhang	1265	0/55	0 (0–6.5)
Northwestern Punjab	Bhakkar	5310	2/56	3.6 (0.4–12.3)
	Khushab	3712	9/55	16.4 (7.8–28.8)
	Mianwali	1886	0/56	0 (0–6.4)
	Sargodha	774	1/55	1.8 (0–9.7)
Southern Punjab	Bahawalnagar	681	0/55	0 (0–6.5)
	Bahawalpur	1078	1/56	1.8 (0–9.6)
	Layyah	3155	0/16	0 (0–20.6)
	Lodhran	115	7/51	13.7 (5.7–26.3)
	Muzaffargarh	1687	0/55	0 (0–6.5)
	Rahimyar Khan	1921	7/56	12.5 (5.2–24.1)
Total		22,943	62/728	8.5 (6.6–10.8)

Prevalence was significantly different among districts, χ^2^ = 114.972, df = 12, *p* < 0.001; prevalence was significantly different among districts zones, χ^2^ = 23.002, df = 2, *p* < 0.001.

**Table 3:** Univariable analysis for *Anaplasma* sp. seropositive camels and the risk-associated factors of Punjab, Pakistan.

Variable	Category	Positive/Tested	Prevalence % (95% CI)	*p*-Value	Odds Ratio	*p*-Value
Geographical location	Central	35/217	16.1 (11.5–21.7)	Chi = 23.002 *p* < 0.01	3.51 (1.87–6.62)	<0.001
	Northern	12/222	5.4 (2.8–9.3)		1.04 (0.48–2.28)	
	Southern	15/289	5.2 (2.9–8.4)		Ref. *	
Season	Summer	60/618	9.7 (7.5–12.3)	χ^2^ = 7.462 *p* = 0.006	5.81 (1.40–24.11)	0.015
	Winter	2/110	1.8 (0.2–6.4)		Ref. *	
Breed	Non-Descript	18/201	8.9 (5.4–13.8)	χ^2^ = 0.098 *p* = 0.952	1.12 (0.55–2.29)	0.952
	Berella	29/341	8.5 (5.8–12)		1.06 (0.55–2.03)	
	Marecha	15/186	8.1 (4.6–13)		Ref. *	
Age/year	>10	10/96	10.4 (5.1–18.3)	χ^2^ = 8.611 *p* = 0.035	2.50 (0.98–6.36)	0.403
	7.1–10	6/100	6 (2.2–12.6)		1.37 (0.47–3.96)	
	3.1–7	37/330	11.2 (8–15.1)		2.71 (1.28–5.74)	
	≤3	9/202	4.5 (2.1–8.3)		Ref. *	
Sex	Female	37/433	8.5 (6.4–11.6)	χ^2^ = 0.001 *p* = 0.973	1.01 (0.59–1.72)	0.973
	Male	25/295	8.5 (5.6–12.3)		Ref. *	
Body condition score	BCS 2	8/12	66.7 (34.9–90.1)	χ^2^ = 55.034 *p* < 0.001	32.0 (8.84–115.86)	<0.001
	BCS 3	35/393	8.9 (6.3–12.2)		1.6 (0.88–2.79)	
	BCS ≥ 4	19/323	5.9 (3.6–9)		Ref. *	
Tick infestation	Yes	51/109	46.8 (37.2–56.6)	χ^2^ = 241.01 *p* < 0.001	48.6 (24.01–98.37)	<0.001
	No	11/619	1.8 (0.9–3.2)		Ref. *	

* Ref.: The lowest value is taken as a reference for comparison. BCS: Body condition score.

**Table 4:** Multivariable analysis for *Anaplasma* sp. seropositive camels and the risk-associated factors of Punjab, Pakistan.

Variable	Exposure	Comparison	Odds Ratio	95% CI	Wald *p*
Geolocation	Central	Southern and Northern	2.78	1.38–5.56	0.004
Season	Summer	Winter	7.94	1.68–37.60	0.009
Body condition score	BCS 2	≥4	14.81	2.03–107.91	0.029
	BCS 3	≥4	1.29	0.63–2.66	
Tick infestation	Yes	No	38.59	18.56–80.22	<0.001

BCS: Body condition score.

## Data Availability

The datasets used and/or analyzed during the current study available from the corresponding author on reasonable request. All data are contained within the manuscript.
